# Pulmonary Veins Morphometric Characteristics and Spatial Orientation Influence on Its Cryoballoon Isolation Results

**DOI:** 10.3390/diagnostics12061322

**Published:** 2022-05-26

**Authors:** Sergey Mamchur, Tatiana Chichkova, Egor Khomenko, Alexander Kokov

**Affiliations:** Federal State Budgetary Institution ‘Research Institute for Complex Issues of Cardiovascular Diseases’, 6, Sosonoviy Blvd., 650002 Kemerovo, Russia; chi4cova@ya.ru (T.C.); homea@kemcardio.ru (E.K.); kokoan@kemcardio.ru (A.K.)

**Keywords:** atrial fibrillation, pulmonary vein anatomy, cryoablation

## Abstract

The aim of this paper is to evaluate the effect of pulmonary vein (PV) morphometric characteristics and spatial orientation on the results of cryoballoon ablation (CBA). Methods: A randomized, prospective, single-center controlled study was conducted, enrolling 230 patients with drug-refractory atrial fibrillation (AF). We compared procedural and long-term outcomes in patients who underwent their first procedure of pulmonary vein isolation (PVI) for AF with either radiofrequency ablation (RFA) (*n* = 108) or CBA (*n* = 122) and assessed their interaction with the different pattern of PV anatomy, morphometric characteristics, and spatial orientation. The primary efficacy endpoint was any documented atrial arrhythmia recurrence (AF, atrial flutter, or atrial tachycardia) lasting over 30 s during a 12-month follow-up after a 90-day blanking period and discontinuation of antiarrhythmic drugs. The procedure’s endpoint was the achievement of PVI. Before the intervention, all patients underwent computed tomography (CT) to assess the PV anatomical variant, maximum and minimum diameters of the PV’s ostia, their cross-sectional area, orifice ovality index, and PV tilt angles. Results: The mean follow-up period was 14 months (12; 24). Long-term efficacy in the cryoablation group was 78.8% and in the RFA group—83.3% (OR = 0.74; 95% CI 0.41–1.3; *p* = 0.31). The RFA results did not depend on PV anatomy. The «difficult» occlusion of the right inferior PV (RIPV) occurred in 12 patients and was associated with a more horizontal PV position in the frontal plane; the mean tilt angle was −15.2 ± 6.2° versus −26.5 ± 6.3° in the absence of technical difficulties (*p* = 0.0001). In 11 cases (9%), during ablation of the right superior PV (RSPV), phrenic nerve injury (PNI) occurred and was associated with the maximum and minimum RSPV diameter, 20.0–20.4 mm (OR = 13.2; 95% CI: 4.7–41.9, *p* < 0.05) and 17.5–20 mm (OR = 12.5; 95% CI 3.4–51, *p* < 0.05), respectively. Patients with arrhythmia recurrence were characterized by significantly larger diameters and ovality of the left superior PV (LSPV). The spatial orientation of the PV does not affect the long-term results of cryoablation. Conclusion: Preprocedural evaluation of PV morphology and orientation using cardiac CT might help choose the optimal technology for the individual patient.

## 1. Introduction

Atrial fibrillation (AF), the most common rhythm disorder, not only affects patients’ quality of life but also worsens their prognosis [1,2,3]. The prevalence of AF increases with age, from 0.7% in the 50–59 age group to 17.8% in the 85 and older group [3,4,5]. AF is associated with a 5.6-fold increase in the risk of ischemic stroke [3,6,7], the development and progression of left ventricular (LV) dysfunction, impaired cognitive function, and an increased risk of sudden cardiac death [3]. The advantages of a conservative strategy for maintaining sinus rhythm are eliminated by the side effects of antiarrhythmic therapy [8]. In 1998, M. Haïssaguerre [9] demonstrated the importance of the ectopic activity of the pulmonary vein (PV) ostia in the initiation of AF. Later, a method of circular isolation was proposed. Achieving the complete electrical isolation of the PV is a cornerstone of AF catheter ablation [1]. To achieve complete PV isolation, it is necessary to create a continuous circular ablation line, ensuring transmural damage. At the same time, the abovementioned expert consensus does not indicate exactly how isolation can be achieved.

For this purpose, radiofrequency ablation (RFA) is the most studied energy. It requires the creation of a continuous line of the block by applying numerous lesions [10]. Although current methods of quantifying RFA, such as the ablation index, allow optimizing ablation parameters for a continuous line of lesions, the effect of cryoenergy on tissue is more «delicate». Cryolesions have smooth, well-demarcated borders and less endocardial thrombus formation compared with RF lesions. There is a «preserving» effect of cryo on collagen, elastic fibers, and microvasculature [11]. The cryoablation method is based on arrhythmogenic zone destruction using deep local cooling along the perimeter of the contact of the cryoballoon with the PV ostium in one single shot. It is recognized as the most promising alternative to radiofrequency ablation. The FIRE and ICE study demonstrated that cryoablation is as effective and safe as RFA [12]. One of the most controversial issues in the cryoballoon technique is the importance of the anatomical factor. In the literature, data on this subject are contradictory and limited, especially in the presence of large PVs [13,14]. Even before the era of the active use of cryotechnology, much work was performed on the imaging of the left atrium (LA). The focus of this work was aimed at the anatomical variant of the PV. Multispiral cardiac computed tomography (CT) is the most widespread for assessing LA anatomy [15,16,17]. Several single-center studies have attempted to identify anatomical predictors of AF recurrence after cryoablation.

The aim of this study was to assess the effect of the PV’s morphometric characteristics and spatial orientation on the efficacy and safety of cryoballoon PV isolation (PVI).

## 2. Materials and Methods

A randomized, prospective, single-center controlled study was conducted, enrolling 230 patients with drug-refractory atrial fibrillation (AF). We compared procedural and long-term outcomes of patients who underwent their first procedure of pulmonary vein isolation (PVI) for AF with either radiofrequency ablation (RFA) (*n* = 108) or CBA (*n* = 122) and assessed their interaction with the different pattern of PV anatomy, morphometric characteristics, and spatial orientation. The study period was between January 2018 and May 2021. The primary efficacy endpoint was any documented atrial arrhythmia recurrence (AF, atrial flutter, or atrial tachycardia) lasting over 30 sec during a 12-month follow-up after a 90-day blanking period and discontinuation of antiarrhythmic drugs. The procedure’s endpoint was the achievement of PVI. Before the intervention, all patients underwent computed tomography (CT) to assess the PV anatomical variant, maximum and minimum diameters of the PV’s ostia, their cross-sectional area, orifice ovality index, and PV tilt angles. Inclusion criteria: age 18–75 years; the presence of paroxysmal or persistent AF, documented on an ECG recording; documented ineffectiveness of antiarrhythmic therapy. Exclusion criteria: prior AF ablation; long-term persistent or permanent AF; the presence of an implanted intracardiac device; chronic obstructive pulmonary disease with signs of the formation of chronic cor pulmonale; left ventricular ejection fraction (EF) <35%; LA diameter ≥55 mm; LA thrombosis; the maximum diameter of one of the PV ≥26 mm.

To study the anatomical features of the LA and PV on a 64-slice tomograph Somatom Sensation 64 (Siemens, Germany), CT angiography of the LA and PV in an ECG-synchronized mode was performed 24 h before the procedure. Contrasting was carried out by intravenous bolus injection of 100 mL of X-ray dye using an automatic syringe-injector into the cubital vein at a rate of 5 mL/s. In all cases, the caudocranial scanning direction was used. The study was performed while the patient was holding their breath. The slice thickness of the obtained axial scans was 1 mm with an interval of 0.9 mm. The analysis of the obtained images was carried out on a Leonardo multimodal workstation (Siemens, Munich, Germany) using a cardiological application included in the manufacturer’s software package. Multiplanar LA images in orthogonal projections, four-chamber position, and images along the LA axes were studied. The presence of thrombotic masses in the heart chambers was determined. Based on the data of PV angiography, their morphometry was performed. The maximum and minimum diameters of their ostia cross-section were estimated, as well as their area. Subsequently, based on these data, the ostial ovality index was calculated. It should be noted that the concept of ‘ostial ovality index’ or its equivalent ‘ostial index’ according to the literature data, does not have a single definition. More often, the calculation of this index is carried out as a ratio of diameters. As it approaches 1, the cross-section is close to the shape of a circle. In other studies, the accepted measure of deviation from the circle is ‘ovality’. Thus, in this work, the ostial index (OI) was calculated by the formula:

$$OI=\frac{2\left(\mathrm{Dmax}-\mathrm{Dmin}\right)}{\mathrm{Dmax}+\mathrm{Dmin}},$$

where Dmax—PV maximal diameter, Dmin—PV minimal diameter.

**Figure 1:**
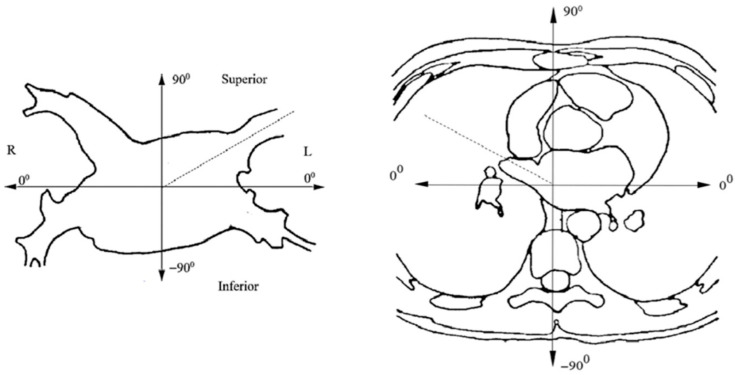
Coordinate system for assessing the PV spatial orientation and the LA posterior wall in the frontal and axial planes.

The spatial orientation of the PV and LA was studied in 2 mutually perpendicular planes—axial (horizontal) and frontal. In the axial plane, the vein deflection angle was determined between the frontal axis, drawn through the center of the PV ostium, and the projection of the vein axis onto the plane. The intersection of the frontal axis with the plane was taken as a reference point. For the convenience of understanding the position of the vein, its posterior deviation was estimated in the range from −90 to 0° and anteriorly—from 0 to 90°, respectively. In the frontal plane, the angle was determined between the horizontal axis drawn through the center of the vein ostium and the projection of the vein axis onto the plane. The upward deflection of the superior PV was assessed in the range from 0 to 90°, of the lower PV—downward from 0° to 90°. For ease of understanding, the standard axis was selected as zero, and the deviation range was from 0° to ±90°. The choice of the coordinate system is demonstrated in Figure 1.

**Figure 2:**
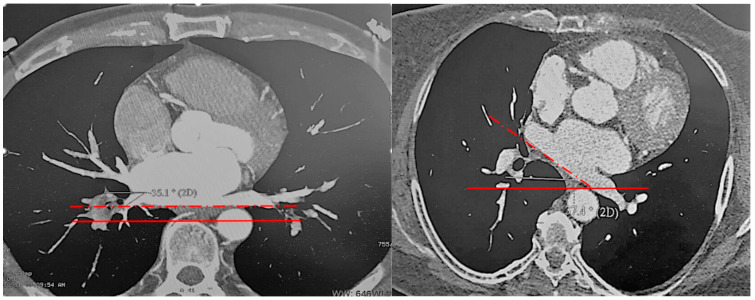
Spatial orientation of the LA posterior wall: red lines—frontal plane; red dot lines—LA posterior wall orientation.

It should be noted that the spatial orientation of the LA posterior wall is variable (Figure 2). In the present study, the angle of its deviation varied from 0 to 45°. Therefore, we recalculated the angles of vein origin in the axial plane relative to the posterior LA wall, which reflects their true spatial orientation.

Cryoballoon PV isolation was performed using balloons of the first and second generations; in the presence of a PV variant anatomy, only balloons of the second generation were used. We performed two 300 s applications per vein using first-generation balloons and one application per vein lasting 240 s using second-generation balloons. If isolation was not achieved, the balloon was rotated by 45 degrees, and additional applications were performed. Phrenic nerve pacing was used for ablation of the right pulmonary veins. In the case of any decrease in the diaphragmatic contraction’s amplitude, the freezing immediately discontinued, and this situation was considered an ‘incomplete’ phrenic nerve palsy. Radiofrequency ablation was performed on the Carto 3 navigation system (Biosense Webster, Irvine, CA, USA) using Smarttouch catheters. The ablation design consisted of only PV antral isolation. In all cases, the presence of an entry and exit block from the PVs was documented.

All patients underwent systematic, standardized follow-up at 3, 6, and 12 months and every 6 months thereafter. A 12-lead ECG and 24-hour Holter ECG were performed at each visit. During the follow-up period, patients visited our hospital any time they felt arrhythmia symptoms, and a 12-lead ECG was recorded. Within the blanking period, recurrent arrhythmia was managed with antiarrhythmic drugs or cardioversion, and repeat ablation was not performed.

Statistical processing of the study results was carried out using the Medcalc v 18.2.1 (Softwa, Ostend, Belgium) and STATISTICA 10 (StatSoft, Tulsa, OK, USA) software packages. Statistical methods for testing hypotheses were selected according to the nature of the distribution of quantitative characteristics, tasks, and data types. Quantitative values were presented as the median and interquartile range (Me (Lq; Uq)) or as the mean and standard deviation (M ± SD). Qualitative values are presented in the form of absolute and relative frequencies. If the distribution did not correspond to the normal distribution, nonparametric tests were used to test statistical hypotheses. A comparison of the two groups was carried out using the Mann–Whitney U test. A comparison of the three unrelated groups was carried out using Kruskal–Wallis rank analysis. To compare qualitative features, the χ2 test with Yate’s correction was used. The level of statistical significance was taken as *p* ≤ 0.05. Control point dynamics were assessed using the Kaplan–Meier analysis.

## 3. Results

**Figure 3:**
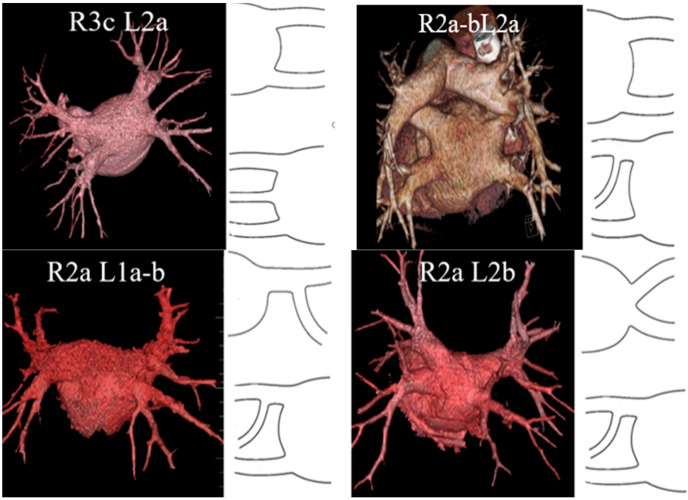
Anatomical variants of PV according to E.M. Marom et al. [18].

According to the initial clinical characteristics and anatomical variants of the PV according to E.M. Marom et al. [18], the groups were comparable (Figure 3, Table 1). ‘Isolated’ (not associated with structural heart disease) AF was present in about 1/3 of cases.

There were no significant differences in the baseline clinical characteristics of the subgroups of the variant and typical anatomy. The incidence of persistent AF in the typical PV anatomy in the cryoablation group was 12.1% (*n* = 12) and in the RFA group—18.4% (*n* = 16), *p* = 0.3; with variant anatomy—6/23 cases in the cryo group and 2/21—In the RFA group, *p* = 0.28; in the presence of a common left PV ostium—3/13 cases in the cryo group and 2/17 in the RFA group with *p* = 0.64.

The prevalence of various PV anatomical variants in the present study was consistent with published data [17,19]. The most common variants were encountered. Long-term results of RFA were not dependent on PV anatomy. Whereas, the presence of a common left PV ostium affected the long-term CBA results (AF freedom was 23.1% versus 84.9% (OR = 0.14; 95% CI 0.03–0.6, *p* = 0.02)). These data were published by us earlier [20]. In this work, we focused on the study of the morphometric characteristics and spatial orientation of the pulmonary veins.

Analysis of intraoperative data did not reveal significant differences in the duration of the left atrial phase of the procedure and the total duration of fluoroscopy. By the end of the intervention, 465/477 (97.5%) and 415/415 (100%) PV were isolated in the cryo and RFA groups, respectively. Table 2 shows the structure of complications. Potentially life-threatening events such as hemopericardium and transient ischemic attacks (TIA) developed in 5 cases. There was 1 case of TIA in the CBA group and 2 cases in the RFA group. Hemopericardium occurred in 1 case in the CBA group (during transseptal puncture) and in 2 cases in the RFA group (during PVI). In all cases, the hemopericardium required drainage.

PNI occurred in 11 cases during the RSPV isolation; in 5 cases, it was persistent. In 6 cases, a transient PNI was identified based on any perceived decrease in the amplitude of diaphragm contraction in response to pacing. The time of PNI in CBA subgroups was 125 ± 41 s and 129 ± 92 s, respectively (*p* = 0.14). PNI was associated with the maximum and minimum RSPV diameter 20.0–20.4 mm (OR = 13.2; 95% CI: 4.7–41.9, *p* < 0.05) and 17.5–20 mm (OR = 12.5; 95% CI 3.4–51, *p* < 0.05), respectively. The cross-sectional area was 2.9 ± 0.55 versus 1.8 ± 0.6 cm^2^ (*p* = 0.001). Nadir temperature was −49 ± 2.3 versus −46.4 ± 2.5 °C (*p* = 0.0012), and temperature to 60 s was −48.9 ± 2.3 versus 46.1 ± 2.6 °C/60 s (*p* = 0.003) in the group with and without PNI, respectively.

**Figure 4:**
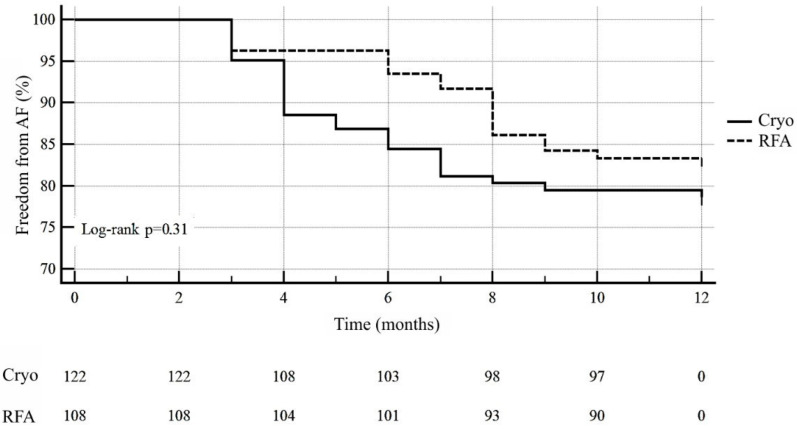
Freedom from AF in cryoballoon and radiofrequency PV’s isolation during 12 months of follow-up.

Mean follow-up period was 14 months (12; 24). Long-term efficacy in the cryoablation group was 78.8%, in the RFA group—83.3% (odds ratio (OR) = 0.74; 95% CI 0.41–1.3; *p* = 0.31) (Figure 4).

The morphometric characteristics of CBA subgroups are shown in Table 3. There were no significant differences between the groups. In the studied sample, the cross-sectional area of the superior PV’s ostia was significantly larger than the lower PV’s: 1.69 versus 1.37 cm^2^ for left PV and 1.86 versus 1.69 cm^2^ for right PV (*p* < 0.0001). At the same time, RSPV was characterized by larger area values than the left superior pulmonary vein (LSPV): 1.86 cm^2^ versus 1.69 cm^2^ (*p* = 0.008). There were no significant differences between the inferior veins (*p* = 0.07). The shape of the right PV’s ostia was more rounded: OI for RSPV and right inferior pulmonary vein (RIPV) was 0.15 in both groups (*p* = 0.82).

Left PVs were characterized by greater ovality (*p* < 0.0001) without significant differences between LSPV and LIPV: 0.33 and 0.3, respectively (*p* = 0.06). The area of the left common trunk was significantly larger than LSPV and LIPV: 3.33 cm^2^ versus 1.69 and 1.37 cm^2^, respectively (*p* < 0.0001). The OI of the common trunk (0.32) corresponded to the trend for the left PV.

In the cryoisolation group, AF recurrence in the long-term follow-up period developed in 26 cases (21.3%)—10 and 16 when using cryoballoons of the first and second generation, respectively. When comparing the group with the AF recurrence with the group free from AF, it was found that the cross-sectional area of the LSPV ostium in the AF recurrence group was higher: 1.69 versus 1.35 cm^2^ (*p* = 0.05). The maximum LSPV diameter was also significantly greater: 18.8 versus 17.5 mm (*p* = 0.048), and the difference in the minimum diameter tended to be significant: 13.4 versus 12.5 mm (*p* = 0.06). Earlier it was shown that the presence of a left common ostium was accompanied by significantly worse long-term results. According to the morphometry data in the arrhythmia recurrence group, the area of the common trunk was 3.39 against 2.88 cm^2^ (*p* = 0.05), the maximum diameter was also larger: 25.4 versus 21.0 mm (*p* = 0.016), and the OI of the common trunk was 0.36 versus 0.18 (*p* = 0.05). No other differences were found.

**Figure 5:**
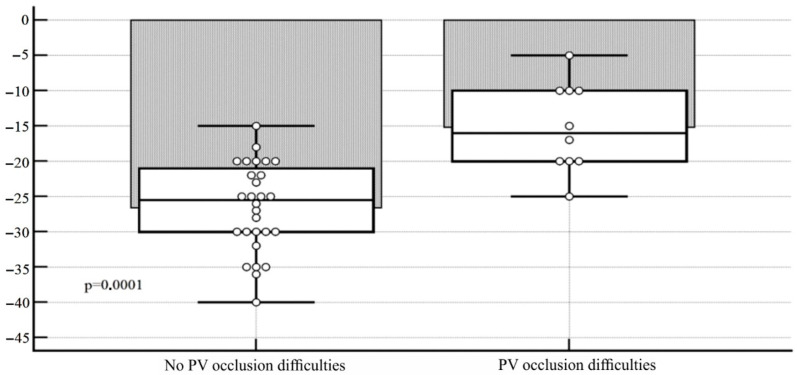
Diagram of angulation ranges of the RIPV in the frontal plane, depending on the presence/absence of technical difficulties in positioning the cryoballoon in its ostium.

**Figure 6:**
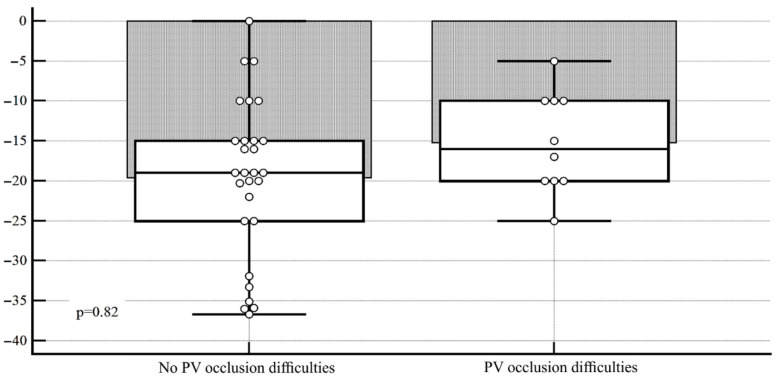
Diagram of angulation ranges of the RIPV in the axial plane, depending on the presence/absence of technical difficulties in positioning the cryoballoon in the ostium.

The use of cryotechnology is associated with technical difficulties in balloon positioning in the RIPV. Occlusion was considered technically difficult if >2 applications were required to achieve PVI, and the velocity of temperature drop was insufficient. Early termination of freezing because of PNI was not «difficult» occlusion. In the present analysis, «difficult» RIPV occlusion arose in 12 cases, while the use of technical maneuvers did not allow achieving vein isolation in only 2 cases. The group in which technical difficulties arose was characterized by a significantly more horizontal position of the vein in the frontal plane: the mean values of the deviation angle were −15.2 ± 6.2 versus −26.5 ± 6.3° (95% CI 6, 6–16.1, *p* = 0.0001). Vein deviation in the axial plane was not statistically different between the groups: −19.6 ± 9.9 versus −18.8 ± 8.7 (95% CI 6.5–8.0, *p* = 0.82). The data are shown in Table 4 and Figure 5 and Figure 6. 

The angulometry data were grouped; for each of the obtained intervals, the likelihood ratio to the development of AF recurrence in the long-term period was calculated. No significant associations were identified (Table 5).

Thus, the spatial orientation of the PV does not affect the long-term results of the PV isolation; however, the horizontal orientation of the PV in the frontal plane creates technical difficulties in achieving the RIPV occlusion, which requires special maneuvers.

## 4. Discussion

Considering the biophysical characteristics of cryoablation, it is likely that in different PV spatial orientations, the pressure of the balloon on the tissue, as well as the intensity of lesion, may be different at different points of contact. Thereafter, it can be assumed that the morphometric characteristics and spatial orientation of the PV’s ostia can influence the outcomes. In the literature, description of this fact is extremely limited. Currently, there is no unified algorithm for estimating the PV’s angles. In the published studies, the measurement methodology, especially the reference point, is not standardized, which creates significant difficulties in interpreting the data and trying to apply them in practice. In some CT studies, an analysis of the PV tilt angle relative to the frontal plane of the body was carried out without considering the LA position. In cryoablation, the significance of this factor has not been studied. However, it is known that the spatial orientation of the PV’s ostia does not affect the outcomes of RFA [21].

When analyzing the results of published works, attention is drawn not only to the inconsistency of the results obtained but also to the fact that the PV’s anatomy was studied mainly from the standpoint of the anatomical variant without analyzing the morphometric and other characteristics of the veins. Several studies have shown a lower efficacy of cryo-isolation in the presence of large PV diameters, especially of RSPV. The studies of the preferred cryoballoon size have also shown that 23 mm devices are less effective.

Thus, we assumed that not only the anatomical variant but also the morphometric parameters of the PV’s ostia are important in the long-term efficacy. Theoretically, with a large-sized PV’s ostia, the lesion is performed more distally from the vein ostium; accordingly, a smaller volume of the antrum myocardium is electrically excluded, which is the main target for ablation. Our data are consistent with the results of other clinical studies. Nanbu T et al. (2018), Yotsukura A, Sano F, et al. studied the relation between ablation area and outcome of ablation using 28-mm CBA depending on morphometric PV’s parameters. The importance of LA and PV anatomy was demonstrated based on bipolar mapping after CBA [22].

In the present study, no significant differences in morphometric characteristics were found between and within the groups. In the studied sample, the cross-sectional area of the superior PV’s ostia was significantly larger than the area of the inferior ones. RSPV was characterized by larger values of the ostium area than LSPV. The shape of the right PV’s ostia was more rounded. Left PVs were characterized by greater ovality without significant differences between LSPV and LIPV. The area of the left common trunk was significantly larger than the left PV. The ostial index of the left common trunk corresponded to the trend for the left PV. In the cryoisolation group, AF recurrence in the long-term follow-up period developed in 21.3% of cases. In the cryoisolation group with typical anatomy of the left PV, the development of recurrence was associated with a larger cross-sectional area of the LSPV ostium. The maximum diameter of the LSPV ostium was also significantly larger, and the difference in the minimum diameter tended to be significant. According to the morphometric data of the left common trunk in the group with arrhythmia recurrence, its area was higher, and the maximum diameter and OI were also larger.

Monitoring the parameters of cryokinetics during ablation allows to indirectly predetermine the effectiveness of PV occlusion. In conditions of a fixed cryoballoon diameter, the anatomical factor can affect the contact area of the balloon’s freezing surface with the atrial wall and its pressure on the tissue. Considering the biophysical characteristics of cryoablation, deliberately transient tissue damage is possible in the zone of the lesion [23].

A group of American researchers followed the path of an integrated approach for the assessment of the anatomical factor and proposed in 2020 a generalized model for assessing the ‘favorable’ and ‘unfavorable’ anatomy of the LA and PV based on CT data [24]. The model included the following factors: oval LSPV and RSPV, large circumference of the RSPV and RIPV ostia, RSPV tilt angle more than 22.7 degrees, atriomegaly (LA diameter more than 4.35 cm), and the presence of an accessory right PV. It should be noted that this study has several limitations, such as small sample size and lack of a control group. The effectiveness of cryoisolation in the Score <4 group and in the Score ≥4 group during 12 months of follow-up was 86 versus 32%, with a significant difference (*p* = 0.001). In this study, the presence of an accessory right PV was a predictor of an unfavorable outcome, while the left common trunk did not affect the result, but the presentation of this variant was low.

## 5. Conclusions

This study elucidated some anatomical characteristics of the PV that might affect CBA results in patients with AF. Evaluation of the PV’s morphology and orientation using preprocedural cardiac CT might help in choosing the optimal technology for the individual patient. Further research might overcome the difficulties in choosing the optimal ablation technology.

## Figures and Tables

**Table 1:** Clinical characteristics of the patients.

Parameter	Cryo (*n* = 122)	RFA (*n* = 108)	*p*
Age, years, Me (Lq; Uq)	57 (53.0; 63.0)	56.5 (52.5; 61.5)	0.57
Gender (male/female), *n* (%)	57 (46.7)/65 (53.3)	57 (52.8)/51 (47.2)	0.88
Body mass index (BMI), kg/m^2^	24.7 (21; 31)	25.0 (22.1; 30,4)	0.77
Arterial hypertension, *n* (%)	86 (70.5)	80 (74.1)	0.65
Myocardial infarction in anamnesis, *n* (%)	15 (12,2)	9 (8.3)	0.44
Percutaneous coronary intervention in anamnesis, *n* (%)	6 (4.9)	5 (4.6)	0.84
NYHA I-II functional class heart failure, *n* (%)	44 (36.1)	47 (43,5)	0.31
‘Isolated’ AF, *n* (%)	38 (31.1)	26 (24.1)	0.30
Transitory ischemic attack/stroke in anamnesis, *n* (%)	10 (8.2)	8 (7.4)	0.98
AF duration, Me (Lq; Uq)	4 (2; 4)	4 (3; 5)	0.64
CHA2DS2Vasc score ≥ 2, *n* (%)	72 (59)	77 (71.3)	0.07
HASBLED score 0–2, *n* (%)	104 (85.2)	95 (88.0)	0.68
Typical PV anatomy (R2a-b L2a)	99 (81.1)	87 (80.6)	0.78
Variant PV anatomy	23 (18.9)	21 (19.4)	0.78
Left PV common trunk (R2a L1a-b)	5 (4.1)	9 (8.3)	0.71
Two ostia of left PV not separated by left atrial wall (R2a L2b)	8 (6.6)	8 (7.4)	0.27
Accessory right PV (R3c L2a)	10 (8.2)	4 (3.7)	0.26

**Table 2:** Complication’s structure.

Parameter	Cryo, *n* = 122	RFA, *n* = 108	*p*
Haemopericardium, *n* (%)	1 (0.8)	1 (1.6)	>0.1
TIA, *n* (%)	1 (0.8)	2 (1.9)	>0.1
Groin complications, *n* (%)	3 (2.5)	5 (4.6)	>0.1
Pericarditis, *n* (%)	6 (4.9)	1 (0.9)	>0.1
PN injury, *n* (%)	11 (9)	-	
Transient PNI, *n* (%)	6 (4.9)	-	-
Persistent PNI, *n* (%)	5 (4.1)	-	
Time to PNI, s	127 ± 67		

Comments. TIA—transient ischemic attack; PNI—phrenic nerve injury.

**Table 3:** Characteristics of cryoablation subgroups according to morphometry and cryokinetic data, Me (Lq; Uq), M ± SD.

Parameter	Cryoballoon Generation						*p*
	Total		1st Generation		2nd Generation		
	*n*	Value	*n*	Value	*n*	Value	
LSPV, maximal diameter, mm	108	17.6 (16.0; 18.4)	58	17.9 (16.4; 18.8)	50	17.5 (15.6; 18.0)	0.09
LSPV, minimal diameter, mm		12.5 (11.5; 14.0)		12.7 (11.5; 14.1)		12.1 (11.2; 13.5)	0.13
LSPV, ostial area, cm^2^		1.69 (1.47; 1.99)		1.78 (1.5; 2.1)		1.66 (1.43; 1.86)	0.10
LSPV, OI		0.32 (0.25; 0.36)		0.32 (0.25; 0.37)		0.32 (0.25; 0.37)	0.94
LIPV, maximal diameter, mm		15.5 (14.5; 17.0)	58	15.5 (14.6; 17)	50	15.6 (13.3; 17)	0.17
LIPV, minimal diameter, mm		11.4 (10.6; 12.8)		11.4 (11; 12)		11.3 (10;13)	0.30
LIPV, ostial area, cm^2^		1.37 (1.20; 1.66)		1.36 (1.26; 1.64)		1.41 (1.05; 1.7)	0.34
LIPV, OI		0.30 (0.24; 0.33)		0.30 (0.25; 0.34)		0.28 (0.20; 0.30)	0.18
RSPV, maximal diameter, mm	120	17.0 (15.4; 18.5)	58	16.8 (15.5; 18.5)	62	16.9 (15; 19)	0.60
RSPV, minimal diameter, mm		14.0 (13.0; 16.0)		14 (13.5; 15.5)		14 (12.6; 16.0)	0.58
RSPV, ostial area, cm^2^		1.86 (1.53; 2.27)		1.87 (1.6; 217.8)		1.86 (1.47; 2.27)	0.59
RSPV, OI	120	0.15 (0.11; 0.21)	58	0.16 (0.13; 0.25)		0.15 (0.10; 0.20)	0.34
RIPV, maximal diameter, mm		16.0 (14.0; 17.5)		15.9 (14.5; 17.5)	62	16 (13.8; 17.5)	0.94
RIPV, minimal diameter, mm		13.5 (12.0; 14.8)		13.5 (12; 14)		13.9 (11.8; 15)	0.65
RIPV, ostial area, cm^2^		1.69 (1.32; 2.00)		1.67 (1.30; 1.98)		1.71 (1.32; 2.03)	0.79
RIPV, OI		0.15 (0.12; 0.22)		0.17 (0.13; 0.24)		0.15 (0.11; 0.26)	0.23
Left common trunk, maximal diameter, mm	13	24.5 (22; 26)	-	-	13	24.5 (22; 26)	-
Left common trunk, minimal diameter, mm		17.25 (16.6; 18.5)		-		17.25 (16.6; 18.5)	-
Left common trunk, ostial area, cm^2^		3.33 (2.91; 3.51)		-		3.33 (2.91; 3.51)	-
Left common trunk, OI		0.32 (0.24; 0.39)		-		0.32 (0.24; 0.39)	-
Right accessory PV, maximal diameter, mm	10	8.0 (6.8; 8.9)	-	-	10	8.0 (6.8; 8.9)	-
Right accessory PV, minimal diameter, mm		6.0 (5.0; 7.7)		-		6.0 (5.0; 7.7)	-
Right accessory PV, ostial area, cm^2^		7.53 (5.3; 1.03)		-		7.53 (5.3; 1.03)	-
Right accessory PV, OI		0.13 (0.06; 0.3)		-		0.13 (0.06; 0.3)	-
LSPV, freeze time, s		-		603 ± 30		276 ± 87.4	<0.0001
LIPV, freeze time, s				601 ± 10		265 ± 74.9	
RSPV, freeze time, s				567 ± 122.7		233 ± 82.1	
LSPV freeze time, s				610 ± 79.4		261 ± 76	
LSPV, T nadir, °C		-		−45 (−46; −43)		−46 (−48; −45)	<0.0001
LIPV, T nadir, °C				−43 (−45; −42)		−42 (−41; −43)	0.06
RSPV, T nadir, °C				−46 (−48; −44)		−48 (−49; −45)	0.02
LSPV, T nadir, °C				−43 (−44; −42)		− 43 (−44; −41)	0.40
LSPV, T at 60 s, °C				−44 (−46; −42)		−46 (−48; −45)	<0.0001
LIPV, T at 60 s, °C				−43 (−45; −42)		−42 (−43; −41)	0.1
RSPV, T at 60 s, °C				−46 (−48; −44)		−48 (−48; −45)	0.04
LSPV, T at 60 s, °C				−41 (−44; −40)		−42 (−44; −39)	0.58

Comments: LSPV—left superior pulmonary vein; LIPV—left inferior pulmonary vein; RSPV—right superior pulmonary vein; LIPV—Left inferior pulmonary vein; OI—ostial index.

**Table 4:** Spatial orientation of PV in second generation cryoballoon.

PV	Deviation Angle in Axial Plane, °	Deviation Angle in Frontal Plane, °
LSPV, *n* = 32	17.3 ± 7.4	18.3 ± 5.3
LIPV, *n* = 32	−13.2 ± 9.6	−17.6 ± 6.8
RSPV, *n* = 39	27.7 ± 7.2	27.6 ± 5.5
RIPV, *n* = 39	−19.4 ± 9.5	−23.6 ± 8.0
Left common trunk, *n* = 7	29.3 ± 3.0	13 ± 6.5

**Table 5:** Influence of PV angulation on long-term results.

PV	Clipping Interval, °	Quantity, *n*	LR+	95% CI
LSPV (axial plane)	0–10	5	1.7	0.2–11
	10–25	21	1.2	0.6–2.1
	25–35	6	0	0.1–8.6
LSPV (frontal plane)	5–20	23	1.1	0.6–1.9
	20–30	9	0.9	0.2–5.3
LIPV (axial plane)	−40–(−20)	8	2.3	0.7–7.8
	−20–(−10)	13	0.6	0–3.6
	−10–0	11	0.7	0.2–4.1
LIPV (frontal plane)	−30–(−20)	15	0.2	0–3.2
	−20–(−10)	15	1.1	0.4–3.1
	−10–0	2	0.2	0–1.5
RSPV (axial plane)	−10–0	1.6	0.7	0.7–3.7
	30–40	0.8	0.4	0.4–1.7
RSPV (frontal plane)	20–25	15	1.05	0.4–2.6
	25–40	24	0.97	0.5–1.7
RLIPV (axial plane)	−40–(−25)	11	0.62	0.2–2.4
	−25–(−10)	24	1.4	0.9–2.2
	−10–0	3	0	0–8.5
RIPV (frontal plane)	−40–(−25)	19	1.0	0.5–2.1
	−25–(−10)	18	0.8	0.3–1.8
	−10–0	1	0.2	0.2–12
Left main trunk (axial plane)	20–35	7	–	–
Left main trunk (frontal plane)	0–10	5	0.2	0.1–1.0
	10–25	2	0.3	0.1–1.0

## Data Availability

The datasets used and analyzed during the current study are available from the corresponding author upon reasonable request.

## References

[1] Calkins H., Hindricks G., Cappato R., Kim Y.H., Saad E.B., Aguinaga L., Akar J.G., Badhwar V., Brugada J., Camm J. (2018). 2017 HRS/EHRA/ECAS/APHRS/SOLAECE expert consensus statement on catheter and surgical ablation of atrial fibrillation. Europace.

[2] Kirchhof P., Benussi S., Kotecha D., Ahlsson A., Atar D., Casadei B., Castella M., Diener H.C., Heidbuchel H., Hendriks J. (2016). 2016 ESC Guidelines for the management of atrial fibrillation developed in collaboration with EACTS. Eur. Heart J..

[3] Hindricks G., Potpara T., Dagres N., Arbelo E., Bax J.J., Blomström-Lundqvist C., Boriani G., Castella M., Dan G.A., Dilaveris P.E. (2021). 2020 ESC Guidelines for the diagnosis and management of atrial fibrillation developed in collaboration with the European Association for Cardio-Thoracic Surgery (EACTS): The Task Force for the diagnosis and management of atrial fibrillation of the European Society of Cardiology (ESC) Developed with the special contribution of the European Heart Rhythm Association (EHRA) of the ESC. Eur. Heart J..

[4] Heeringa J., van der Kuip D.A., Hofman A., Kors J.A., van Herpen G., Stricker B.H., Stijnen T., Lip G.Y., Witteman J.C. (2006). Prevalence, incidence and lifetime risk of atrial fibrillation: The Rotterdam study. Eur. Heart J..

[5] Go A.S., Hylek E.M., Phillips K.A., Chang Y., Henault L.E., Selby J.V., Singer D.E. (2001). Prevalence of diagnosed atrial fibrillation in adults: National implications for rhythm management and stroke prevention: The AnTicoagulation and Risk Factors in Atrial Fibrillation (ATRIA) Study. JAMA.

[6] Wolf P.A., Abbott R.D., Kannel W.B. (1987). Atrial fibrillation: A major contributor to stroke in the elderly. The Framingham Study. Arch. Intern. Med..

[7] Romero J.R., Wolf P.A. (2013). Epidemiology of Stroke: Legacy of the Framingham Heart Study. Glob. Heart.

[8] Wyse D.G., Waldo A.L., DiMarco J.P., Domanski M.J., Rosenberg Y., Schron E.B., Kellen J.C., Greene H.L., Mickel M.C., Dalquist J.E. (2002). A comparison of rate control and rhythm control in patients with atrial fibrillation. N. Engl. J. Med..

[9] Haïssaguerre M., Jaïs P., Shah D.C., Takahashi A., Hocini M., Quiniou G., Garrigue S., Le Mouroux A., Le Métayer P., Clémenty J. (1998). Spontaneous initiation of atrial fibrillation by ectopic beats originating in the pulmonary veins. N. Engl. J. Med..

[10] Cappato R., Calkins H., Chen S.A., Davies W., Iesaka Y., Kalman J., Kim Y.H., Klein G., Natale A., Packer D. (2009). Prevalence and causes of fatal outcome in catheter ablation of atrial fibrillation. J. Am. Coll. Cardiol..

[11] Khairy P., Dubuc M. (2008). Transcatheter cryoablation part I: Preclinical experience. Pacing Clin. Electrophysiol..

[12] Kuck K.H., Fürnkranz A., Chun K.R., Metzner A., Ouyang F., Schlüter M., Elvan A., Lim H.W., Kueffer F.J., Arentz T. (2016). Cryoballoon or radiofrequency ablation for symptomatic paroxysmal atrial fibrillation: Reintervention, rehospitalization, and quality-of-life outcomes in the FIRE AND ICE trial. Eur. Heart J..

[13] Khoueiry Z., Albenque J.P., Providencia R., Combes S., Combes N., Jourda F., Sousa P.A., Cardin C., Pasquie J.L., Cung T.T. (2016). Outcomes after cryoablation vs. radiofrequency in patients with paroxysmal atrial fibrillation: Impact of pulmonary veins anatomy. Europace.

[14] Heeger C.H., Tscholl V., Wissner E., Fink T., Rottner L., Wohlmuth P., Bellmann B., Roser M., Mathew S., Sohns C. (2017). Acute efficacy, safety, and long-term clinical outcomes using the second-generation cryoballoon for pulmonary vein isolation in patients with a left common pulmonary vein: A multicenter study. Heart Rhythm.

[15] Gebhard C., Krasniqi N., Stähli B.E., Klaeser B., Fuchs T.A., Ghadri J.R., Haegeli L., Lüscher T.F., Kaufmann P.A., Duru F. (2014). Characterization of Pulmonary Vein Dimensions Using High-Definition 64-Slice Computed Tomography prior to Radiofrequency Catheter Ablation for Atrial Fibrillation. Cardiol. Res. Pract..

[16] Hauser T.H., Peters D.C., Wylie J.V., Manning W.J. (2008). Evaluating the left atrium by magnetic resonance imaging. Europace.

[17] Krum D., Hare J., Gilbert C., Choudhuri I., Mori N., Sra J. (2013). Left Atrial Anatomy in Patients Undergoing Ablation for Atrial Fibrillation. J. Atr. Fibrillation.

[18] Marom E.M., Herndon J.E., Kim Y.H., McAdams H.P. (2004). Variations in pulmonary venous drainage to the left atrium: Implications for radiofrequency ablation. Radiology.

[19] Heist E.K., Holmvang G., Abbara S., Ruskin J.N., Mansour M. (2008). Pre-Procedural Imaging to Direct Catheter Ablation of Atrial Fibrillation: Anatomy and Ablation Strategy. J. Atr. Fibrillation.

[20] Chichkova T.Y., Mamchur S.E., Khomenko E.A., Romanova M.P., Kokov A.N. (2019). Impact of pulmonary vein anatomy on cryoballoon isolation results. Complex Issues Cardiovasc. Dis..

[21] Gal P., Ooms J.F., Ottervanger J.P., Smit J.J.J., Adiyaman A., Misier A.R.R., Delnoy P.P.H., Jager P.L., Elvan A. (2015). Pulmonary vein orientation assessment: Is it necessary in patients undergoing contact force sensing guided radiofrequency catheter ablation of atrial fibrillation. Int. J. Cardiol. Heart Vasc..

[22] Nanbu T., Yotsukura A., Sano F., Suzuki G., Ishidoya Y., Yoshida I., Sakurai M. (2018). A relation between ablation area and outcome of ablation using 28-mm cryoballon ablation: Importance of carina region. J. Cardiovasc. Electrophysiol..

[23] Bredikis A., Wilber D. (2011). Factors that Determine Cryolesion Formation and Cryolesion Characteristics. Cryoablation of Cardiac Arrhythmias.

[24] Vaishnav A.S., Alderwish E., Coleman K.M., Saleh M., Makker P., Bhasin K., Bernstein N.E., Skipitaris N.T., Mountantonakis S.E. (2020). Anatomic predictors of recurrence after cryoablation for atrial fibrillation: A computed tomography based composite score. J. Interv. Card. Electrophysiol..

